# Changes of liver metabolites following hepatectomy with ischemia reperfusion towards liver regeneration

**DOI:** 10.1002/ags3.12058

**Published:** 2018-01-30

**Authors:** Yu Saito, Yuji Morine, Shuichi Iwahashi, Tetsuya Ikemoto, Satoru Imura, Hisami Yamanaka‐Okumura, Akiyoshi Hirayama, Tomoyoshi Soga, Masaru Tomita, Mitsuo Shimada

**Affiliations:** ^1^ Department of Surgery Tokushima University Tokushima Japan; ^2^ Department of Clinical Nutrition and Food Management Tokushima University Tokushima Japan; ^3^ Institute for Advanced Biosciences Keio University Tsuruoka Japan

**Keywords:** ischemia reperfusion, liver regeneration, metabolome analysis, tryptophan, valine

## Abstract

**Background:**

Metabolome analysis is one of the omics which investigates the final product of a central dogma. Changes of liver metabolites during liver regeneration following hepatectomy (Hx) continue to remain unclear. The aim of the present study was to investigate the changes of liver metabolites following Hx with ischemia reperfusion (I/R) towards liver regeneration.

**Methods:**

Twenty‐three patients who underwent Hx were enrolled in this study. Non‐tumor tissues were sampled immediately before and after Hx and a comparison was made between the liver samples taken before and after Hx using capillary electrophoresis (CE)—time‐of‐flight mass spectrometry (TOFMS) as metabolome analysis.

**Results:**

The metabolic pathway showed that there was a significant increase in “lactate” following Hx. There was a significant decrease in metabolites only in the first half of the tricarboxylic acid cycle (TCA) cycle, and adenosine triphosphate (ATP) by anaerobiotic glycolysis did not occur in time for energy consumption of the Hx. Principal component analysis revealed remarkably different component profiles between the samples taken before and after Hx. One hundred and three metabolites were selected as critical metabolites for separating components. Valine and tryptophan increased significantly after Hx and they were regulated by resected liver volume, ischemic time and liver function.

**Conclusion:**

The liver metabolites changed remarkably between before and after Hx. Especially, liver valine and tryptophan were increased.

## 1 INTRODUCTION

Recently, focus has been on metabolome analysis as a method of post‐genomic analysis. Metabolome analysis is one of the omics, which investigates the final product of a central dogma. Metabolome analysis is defined as the comprehensive analysis of small molecules (typically <1.5 kDa) such as amino acids, organic acids, sugars, lipids, inorganic ions and so on.^1^ The number of human metabolites is estimated to range from 2500 to 8000.^2^, ^3^ Currently, three major analytical methods have been widely used in metabolome analysis including gas chromatography/mass spectrometry (GC/MS), liquid chromatography‐mass spectrometry (LC‐MS) and capillary electrophoresis‐mass spectrometry (CE‐MS), and each method is able to analyze a different class of metabolites.^3^, ^4^

It has been well established that the liver can regenerate after hepatectomy (Hx).^5^ The authors have previously reported that liver regeneration is a fundamental mechanism by which the liver responds to various injuries such as ischemia reperfusion (I/R) injury.^6^, ^7^, ^8^ Although the molecular and cellular mechanism of liver regeneration after Hx is well understood, postoperative liver failure after Hx is still one of the critical problems in clinical settings.

There have been no reports about liver metabolites during liver regeneration using metabolome analysis. It was hypothesized that some key metabolites immediately after Hx could regulate or stimulate future liver regeneration. The aim of the present study was to investigate changes of liver metabolites following Hx with I/R towards liver regeneration using human Hx samples.

## 2 MATERIALS AND METHODS

### 2.1 Patients

Twenty‐three patients who underwent Hx between April 2014 and March 2015 were enrolled in this study. Inclusion criteria for this study were: (i) primary Hx; (ii) <15% of indocyanine green retention test (ICG R15); (iii) subsegmentectomy or more extended Hx; (iv) no biliary reconstruction and lymph node dissection; and (v) no preoperative chemotherapy. Backgrounds of the patients are shown in Table 1. The study was approved by the Tokushima University Hospital Ethics Committee and the corresponding regulatory agencies and all the experiments were carried out in accordance with the approved guidelines. Meanwhile, all the patients involved in the study signed the informed consent form and agreed to participate (To CMS ID; 1815).

**Table 1:** Backgrounds of 23 patients who underwent Hx between April 2014 and March 2015

Gender	
Male/Female	20/3
Age y (range)	65 (36‐81)
Hepatitis virus	
HBV/HCV/nBnC	7/7/9
Primary disease	
HCC/CCC/CRLM/Others)	19/1/1/2
Operative procedures	
HrS/Hr1/Hr2	7/4/12
Resected liver volume (%/total liver volume)	
HrS/Hr1/Hr2	9 (6‐12)/23 (20‐28)/52 (44‐61)
Operative time min (range)	314 (223‐452)
Ischemic time min (range)	42 (25‐81)
Blood loss mL (range)	175 (40‐644)

CCC, cholangiocellular carcinoma; CRLM, colorectal liver metastases; HBV, hepatitis B virus; HCV, hepatitis C virus; HCC, hepatocellular carcinoma; HrS, hepatic subsegmentectomy; Hr1, hepatic segmentectomy; Hr2, hepatic lobectomy; Hx, hepatectomy; nBnC, non B non C hepatitis.

### 2.2 Sample collection

Non‐tumor tissues were sampled immediately before and after Hx. The collected samples were quickly frozen at −80°C until sample preparation was completed.

### 2.3 Metabolome analysis

Frozen tissue (c.a. 40 mg) was added to methanol (500 μL) containing internal standards (20 μmol L^−1^ each of methionine sulfone and d‐camphor‐10‐sulfonic acid) and homogenized using a beads beater (TOMY Micro Smash MS‐100R; Tomy Digital Biology, Tokyo, Japan) at 3000 rpm for 60 seconds. Then, both chloroform (500 μL) and Milli‐Q water (200 μL) were added to the homogenate. The solution was thoroughly mixed, then centrifuged at 4600 *g* for 15 minutes at 4°C, and the aqueous fraction was centrifugally filtered through a 5‐kDa‐cut‐off ultra‐centrifugal filter unit (Ultrafree‐MC‐PLHCC‐HMT; Human Metabolome Technologies Inc., Tsuruoka, Japan) to remove proteins. The filtrate was dried using an evacuated centrifuge and dissolved in Milli‐Q water (50 μL) containing 200 μmol L^−1^ reference compounds (3‐aminopyrrolidine and trimesic acid) prior to CE‐MS analysis. CE‐MS‐based metabolomic profiling and data analysis were carried out essentially as described.^9^, ^10^, ^11^, ^12^, ^13^

### 2.4 Definitions of clinical parameters

Preoperative, postoperative and changes between pre‐ and postoperative valine and tryptophan were compared in terms of the following clinical parameters (resected liver volume, regeneration rate, ischemic time, FIB‐4 index, and sarcopenia).

1. Resected liver volume: subsegmentectomy was grouped for the minor Hx group, and segmentectomy and lobectomy were grouped for the major Hx group.
2. Regeneration rate: liver volumes before Hx and 1 week after Hx were measured using a 3D simulation imaging system. Regeneration rate was defined as the volume increase of the remnant liver as compared with the preoperative volume. Calculations were made using the following equation: regeneration rate = ([postoperative liver volume] − [preoperative liver volume]/[preoperative liver volume]) × 100 (%). The regeneration rate was divided into low and high regeneration groups by median value.
3. Ischemic time: the cut‐off value of Pringle time was set at 30 minutes.
4. FIB‐4 index: the cut‐off index value was set at 1.50.
5. Sarcopenia: this was defined as both low grip strength and low muscular mass.

Low grip strength was defined as <26 kg, male or <18 kg, female.^14^ Muscular mass was examined with InBody 770^®^ (Kotoku, Tokyo, Japan). Low muscular mass was defined as <90% of the standard (ranges from 90% to 110% of the standard) obtained by the InBody 770^®^.^15^

### 2.5 Statistical analysis

All data are expressed as median (range). Statistical analysis was carried out using Prism 6.07 for Windows (GraphPad Software Inc., La Jolla, CA, USA). *P*‐values <.05 were considered to indicate statistically significant differences. Values between before and after Hx were compared using the Wilcoxon matched‐pairs signed‐rank test. Heat maps of metabolite levels were generated using hierarchical clustering based on Pearson correlation coefficients using the MultiExperiment Viewer (MeV) software (Institute for Genomic Research, Rockville, MD, USA). The data were exported and analyzed by principal components analysis (PCA) using SIMCA‐P software 12.0.1 (Umetrics AB, Umea, Sweden) to visualize the metabolic changes between preoperative and postoperative patients after mean centering and unit variance scaling.

## 3 RESULTS

### 3.1 Metabolic map

**Figure 1:**
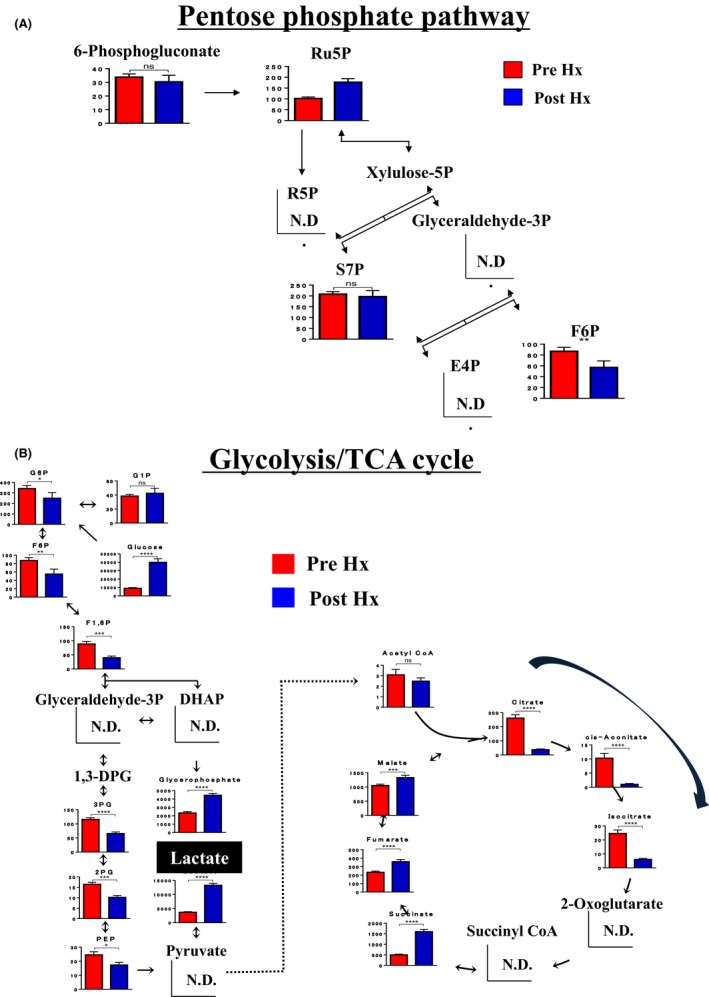
Metabolic map during liver regeneration. A, Pentose phosphate pathway; there were no significant differences between before and after hepatectomy (Hx). B, Glycolysis/tricarboxylic acid cycle (TCA) cycle; there was significant decrease only in metabolites in the first half of the TCA cycle, and ATP by anaerobiotic glycolysis did not occur in time for energy consumption of Hx. N.D., not detected

In the pentose phosphate pathway, there were no significant differences between before and after Hx (Figure 1A). The metabolic pathway showed that there was a significant increase in “lactate” after Hx. There was only a significant decrease in metabolites in the first half of the TCA cycle, and adenosine triphosphate (ATP) by anaerobiotic glycolysis did not occur in time for energy consumption of the Hx (Figure 1B). This suggested that lipid metabolism might be more dominant than glucose metabolism after Hx.

### 3.2 Principal components analysis

**Figure 2:**
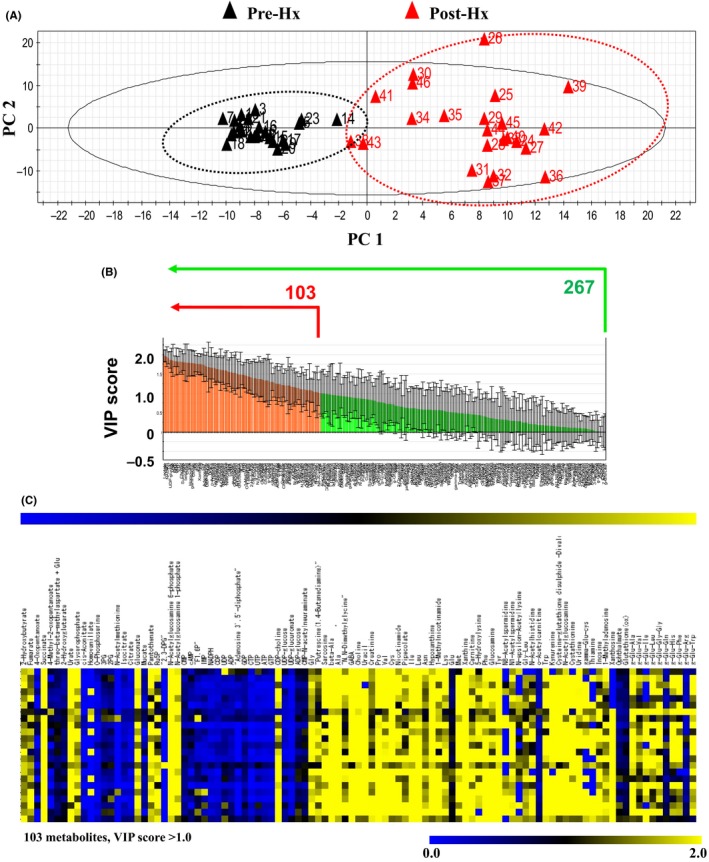
A, Principal components analysis (PCA); PCA shows remarkably different components between before and after hepatectomy (Hx). B, Key 103 metabolites (VIP >1.0); 103 metabolites in a total of 267 metabolites that had a VIP score of more than 1.0 were selected as critical metabolites for separating components. C, Hierarchical clustering. VIP, variable importance for projection

Principal components analysis showed remarkably different component profiles between before and after Hx (Figure 2A). Out of a total of 267 metabolites, 103 metabolites that had a variable importance for projection (VIP) score of more than 1.0 were selected as critical metabolites for separating components (Figure 2B). Table 2 shows all metabolites (VIP score >1.0) and Figure 2C shows hierarchical clustering of 103 metabolites. Valine (VIP score: 1.78957) and tryptophan (VIP score: 1.74943) were significantly up‐regulated after Hx.

**Table 2:** Metabolites for separating components (VIP >1.0)

	Metabolites	VIP score		Metabolites	VIP score
1	Lactate	1.97604	53	UTP	1.4773
2	CDP	1.96398	54	1‐Methyladenosine	1.46181
3	NADPH	1.94671	55	*O*‐Phosphoserine	1.45222
4	Tyr	1.91601	56	1‐Methylnicotinamide	1.44062
5	UDP‐glucose	1.86349	57	Met	1.4312
6	Ala	1.85245	58	2‐Hydroxybutyrate	1.41113
7	ADP	1.8519	59	cis‐Aconitate	1.39913
8	GTP	1.82565	60	g‐Glu‐Ile	1.39354
9	GDP	1.82553	61	Cys	1.38865
10	CTP	1.82183	62	*N*‐Acetylglucosamine	1.37152
11	Leu	1.8197	63	Inosine	1.36748
12	Choline	1.81496	64	Carnitine	1.36734
13	Succinate	1.80697	65	Gluconate	1.35793
14	ATP	1.80538	66	2,3‐DPG	1.34099
15	beta‐Ala	1.80077	67	Glutathione(ox)	1.33471
16	Urate	1.79285	68	2PG	1.33419
17	Val	1.78957	69	g‐Glu‐Arg	1.3306
18	Homovanillate	1.78688	70	Uridine	1.29395
19	gamma‐Glu‐cys	1.78348	71	g‐Glu‐Ala	1.29336
20	Citrate	1.78155	72	5‐Hydroxylysine	1.29306
21	Ile	1.77265	73	g‐Glu‐Gly‐Gly	1.28145
22	Pro	1.76593	74	2‐Hydroxyglutarate	1.25326
23	Phe	1.76262	75	ADP‐glucose	1.24392
24	Xanthine	1.75003	76	IMP	1.2395
25	Trp	1.74943	77	F1,6P	1.2363
26	Gly	1.74604	78	Creatinine	1.21506
27	Adenosine 3′,5′‐diphosphate	1.7337	79	CMP‐*N*‐acetylneuraminate	1.21174
28	Kynurenine	1.71002	80	g‐Glu‐Trp	1.20549
29	UDP‐glucuronate	1.69116	81	Sarcosine	1.2001
30	CMP	1.67643	82	Fumarate	1.19877
31	Uracil	1.66857	83	4‐Methyl‐2‐oxopentanoate	1.19797
32	*o*‐Acetylcarnitine	1.66437	84	Ru5P	1.17545
33	Thiamine	1.66161	85	Mucate	1.16441
34	Glycerophosphate	1.6597	86	*N*‐Acetylhistidine	1.16288
35	*N*‐Acetylglucosamine 6‐phosphate	1.63273	87	g‐Glu‐Phe	1.1496
36	Isocitrate	1.62491	88	g‐Glu‐Leu	1.13668
37	Glucose	1.62172	89	g‐Glu‐Val	1.13623
38	4‐Oxopentanoate	1.60649	90	Gly‐Leu	1.11075
39	Hypoxanthine	1.6039	91	*N*,*N*‐Dimethylglycine	1.10164
40	Nicotinamide	1.59889	92	g‐Glu‐His	1.09598
41	Xanthosine	1.59198	93	Cysteine‐glutathione disulfide ‐ divalent	1.09562
42	*N*‐Acetylglucosamine 1‐phosphate	1.58238	94	Ophthalmate	1.08533
43	Lys	1.55611	95	*N*‐Acetylmethionine	1.0849
44	GABA	1.5491	96	Pipecolate	1.08293
45	cAMP	1.54597	97	Pantothenate	1.07741
46	threo‐beta‐methylaspartate + Glu	1.53009	98	Putrescine (1,4‐butanediamine)	1.06563
47	CDP‐choline	1.52931	99	g‐Glu‐Gln	1.0381
48	Cystathionine	1.52375	100	*N*‐epsilon‐acetyllysine	1.03715
49	*N*1‐Acetylspermidine	1.50057	101	Glu	1.03233
50	Glucosamine	1.48626	102	*N*8‐Acetylspermidine	1.01626
51	3PG	1.48145	103	UDP	1.00423
52	Asn	1.48075			

VIP, variable importance for projection.

### 3.3 Valine/tryptophan and clinical parameters

In the case of valine, much more resected volume and longer ischemic time affected its up‐regulation. Furthermore, preoperative valine significantly increased in the high FIB‐4 group whereas sarcopenia did not affect valine expression. Tryptophan was found to have similar tendencies to valine (Table 3).

**Table 3:** Valine/tryptophan and clinical parameters

	Grouping	Valine	*P*‐value
Resected LV			
Post/Pre	HrS vs Hr1 or Hr2	1.9 (1.2‐2.3) vs 2.4 (1.4‐4.1)	*P* < .01
Liver RR	Cut‐off; median		
Post/Pre	Low vs High	2.4 (1.2‐2.7) vs 2.2 (1.4‐4.1)	*P* = .72
Ischemic time	Cut‐off; 30 min		
Post/Pre	Short vs Long	1.5 (1.2‐2.3) vs 2.3 (1.4‐4.1)	*P* = .08
FIB‐4 index	Cut‐off; 1.50		
Pre	Low vs High	199 (142‐307) vs 258 (187‐458)	*P* = .04
Post		498 (374‐614) vs 568 (322‐927)	*P* = .21
Post/Pre		2.3 (1.9‐3.3) vs 2.3 (1.2‐4.1)	*P* = .41
Sarcopenia			
Pre	Non‐Sarco. vs Sarco.	238 (142‐458) vs 277 (224‐291)	*P* = .99
Post		571 (374‐927) vs 558 (322‐617)	*P* = .50
Post/Pre		2.3 (1.2‐4.1) vs 1.9 (1.4‐2.2)	*P* = .22

HrS, hepatic subsegmentectomy; Hr1, hepatic segmentectomy; Hr2, hepatic lobectomy; LV, liver volume; RR, regeneration rate; Sarco., sarcopenia.

## 4 DISCUSSION

In the present metabolomics study: (i) the metabolic pathway showed that lipid metabolism might be more dominant than glucose metabolism after Hx; (ii) liver metabolites changed remarkably between before and after Hx; and (iii) liver valine and tryptophan were remarkably increased after Hx and they were regulated by resected liver volume, ischemic time and liver function.

It has already been reported that remnant liver metabolism after Hx switches to a predominant utilization of fatty acid as an energy source from glucose.^16^, ^17^ Within 24 hours after Hx, the uptake of free fatty acids in the residual hepatocytes was increased in order to acquire energy, and hepatocytes accumulated triglyceride (TG).^18^, ^19^, ^20^ In the present study, glucose metabolism did not occur in time for energy consumption after Hx, and lipid metabolism might be dominant for producing ATP. These results were reasonable for previous reports.

Regarding liver metabolism during liver regeneration, there was a report on amino acid and glucose metabolism in the rat fulminant hepatic failure (FHF) model.^21^ In that model, a new isolated perfused liver system in combination with a mass‐balance model to generate a metabolic map was introduced. However, the number of metabolites was limited, and it was not a comprehensive analysis. Conversely, metabolome analysis is concerned with the comprehensive analysis of endogenous low‐molecular‐weight compounds in biological samples.

Valine, which is one of the branched‐chain amino acids (BCAA), has been reported to stimulate the proliferation of hepatocytes by a dose dependent method in vitro.^22^ Valine was also found to be most effective in vivo among three BCAA in the Hx model. Furthermore, valine was reported to increase serum free fatty acid, or liver triglyceride, and up‐regulated liver fatty acid became a source of ATP production.^22^ Valine, was also reported to have an antioxidative effect by down‐regulating tumor necrosis factor (TNF) and up‐regulating superoxide dismutase 2 (SOD2) towards human umbilical vein endothelial cells (HUVEC).^23^

In contrast, tryptophan was the major source of serotonin production, and platelets were major carriers of serotonin in the blood. In thrombocytopenic mice, a serotonin agonist reconstituted liver proliferation after Hx.^24^ Tryptophan might stimulate liver regeneration after Hx by serotonin. Furthermore, tryptophan regulated reactive oxygen species (ROS) by inducing nuclear factor (erythroid‐derived 2)‐like 2 (NF‐E2‐related factor 2 or Nrf2) in primary hepatocyte culture.^25^ In our hierarchical clustering, valine and tryptophan significantly increased after Hx, and they were regulated by resected liver volume, ischemic time and liver function. Although further investigations are necessary, these phenomena might reflect the remnant liver's protective response for I/R injury.

It was already reported that the period immediately after Hx (0‐6 hours after Hx) was critical for future liver regeneration.^5^, ^6^ In the present study, liver tissues were sampled immediately after Hx, and we hypothesized that metabolites immediately after Hx would affect future liver regeneration. However, there was a limitation of this study regarding the time‐point after Hx, and several time‐points after Hx might be necessary.

In conclusion, the present study identified the changes of liver metabolites in Hx with I/R towards liver regeneration. Liver metabolites changed remarkably between before and after Hx. In particular, liver valine and tryptophan were increased after Hx.

## DISCLOSURE

Conflicts of Interest: Authors declare no conflicts of interest for this article.

Ethical Approval: All procedures carried out in studies involving human participants were in accordance with the ethical standards of the institutional and/or national research committee and with the 1964 Helsinki declaration and its later amendments or comparable ethical standards.

Informed Consent: Informed consent was obtained from all individual participants included in the study.
